# Bacterial vaginosis testing gaps for transmasculine patients may exacerbate health disparities

**DOI:** 10.3389/frph.2024.1344111

**Published:** 2024-02-20

**Authors:** Hale M. Thompson, Laura K. Rusie, John A. Schneider, Supriya D. Mehta

**Affiliations:** ^1^ Center for Education, Research & Advocacy, Howard Brown Health, Chicago, IL, United States; ^2^ Division of Infectious Disease Medicine, Rush University Medical Center, Chicago, IL, United States; ^3^ Chicago Center for HIV Elimination, Department of Infectious Disease, The University of Chicago, Chicago, IL, United States

**Keywords:** gender-affirming care, vaginal microbiome, bacterial vaginosis, testosterone, health disparities, STI, transgender, transmasculine

## Abstract

**Introduction:**

Bacterial vaginosis (BV) is associated with non-optimal changes in the vaginal microbiome and increased susceptibility to STIs and HIV in cisgender women. Much less is known about the sexual health of transmasculine people and susceptibility to BV, STIs, and HIV. This study's objective was to assess BV testing and outcomes of transmasculine and cisgender women patient populations at a large, LGBTQ + federally qualified health center.

**Methods:**

Retrospective electronic health record data were extracted for eligible patients having at least one primary care visit between January 1, 2021, and December 31, 2021. Transmasculine patients were limited to those with a testosterone prescription in 2021. We conducted log binomial regression analysis to determine the probability of receiving a BV test based on gender identity, adjusting for sociodemographic characteristics.

**Results:**

During 2021, 4,903 cisgender women patients and 1,867 transmasculine patients had at least one primary care visit. Compared to cisgender women, transmasculine patients were disproportionately young, White, queer, privately insured, living outside Chicago, and had a lower rate of BV testing (1.9% v. 17.3%, *p* < 0.001). Controlling for sociodemographics, transmasculine patients were less likely to receive a BV test [Prevalence Ratio = 0.19 (95% CI 0.13–0.27)].

**Discussion:**

The low rate of BV testing among transmasculine patients may contribute to disparities in reproductive health outcomes. Prospective community- and provider-engaged research is needed to better understand the multifactorial determinants for sexual healthcare and gender-affirming care for transmasculine patients. In particular, the impact of exogenous testosterone on the vaginal microbiome should also be determined.

## 1 Introduction

Bacterial vaginosis (BV) is one of the most common reproductive tract infections, associated with non-optimal changes in the vaginal microbiome (VMB). It is characterized by a dominance of anaerobic bacteria and a reduction in *Lactobacillus crispatus*, which dominates an optimal VMB (1). Decreasing levels of endogenous estrogen are associated with BV (2, 3). Studies among cisgender (see Nomenclature section for definition) women have established that BV and a sub-optimal VMB are associated with adverse outcomes like sexually transmitted infections (STI), HIV, pelvic inflammatory disease, miscarriage, and pre-term birth (4–7). BV raises additional clinical and public health concerns, being associated with stress, anxiety, and uncomfortable symptoms (8).

Far less is known about the sexual and reproductive health of transmasculine individuals (see Nomenclature section) who face numerous barriers to healthcare, including testing for HIV, STIs, and BV. Compared to cisgender women, transmasculine people are less likely to have received any HPV testing or screening for cervical cancer (9). In a global health survey, transgender men (see Nomenclature section) who have sex with men (TMSM) had a significantly lower odds of reporting access to lubricants (OR = 0.54, 95% CI = 0.30, 0.98) and HIV testing (OR = 0.57, 95% CI = 0.33, 0.98) compared to cisgender age- and race-matched controls (10). Similarly, national survey data reveal testing disparities: after controlling for demographic traits in the Behavioral Risk Factor Surveillance System survey conducted in 27 states plus Guam, transmasculine respondents had a lower prevalence of ever testing for HIV (32% vs. 62%) or past year HIV testing (10% vs. 22%) compared to cisgender gay and bisexual men (11). A 2017 cross-sectional online survey of MSM, including 192 TMSM, found that approximately 30% of TMSM reported never having had a viral or bacterial STI test (12). A study derived from 2016 to 2020 testing data at a Seattle sexual health clinic found that transgender men had a high prevalence of rectal chlamydia and syphilis (9% and 5%), and that asymptomatic transgender men were more likely to receive extragenital gonorrhea and chlamydia screening than nonbinary patients assigned female at birth (13).

Shifting from social determinants to biological or biobehavioral ones, very little is known about the impact of gender-affirming medical interventions on transmasculine populations’ sexual health, in particular, the effect of exogenous testosterone therapy on the VMB (14–17). Transmasculine people who use exogenous testosterone typically experience decreasing levels of endogenous estrogen (18, 19). To date, only one study has compared testosterone-dominated VMB of transmasculine adults to VMB of cisgender women. In a small cross-sectional study, 89% (25/28) of transmasculine VMB were not dominated by *Lactobacillus* species whereas 100% (*n* = 8) of cisgender women's VMB were dominated by *Lactobacillus*; additionally, there were greater serum testosterone concentrations among transmasculine participants (all within or slightly above adult cisgender men reference interval) compared to cisgender women participants (all within the adult female reference interval; *p* < 0.001) despite no difference in estrogen concentrations (*p* = 0.84) (15). In a chart review study, Lin et al. compared cervical cytology of 61 transmasculine patients to institutional data of cisgender women and a cohort of cisgender women with vaginal atrophy. There were disproportionately higher rates of unsatisfactory cytology results for transmasculine individuals: 16% compared to 2% among institutional data and atrophic cisgender women, with higher rates of high grade squamous intraepithelial lesions among transmasculine (3%) than institutional data (0.3%) and cisgender women with vaginal atrophy (0%) (20). Cytology slides were available for 46 transmasculine individuals, and *Lactobacilli* were “substantially decreased” in 89%. Authors also reported that “patients with near absence of *Lactobacilli*” had longer duration of gender-affirming hormone therapy compared to those with presence of *Lactobacilli* (mean 5.9 vs. 1.8 years, *p* = 0.017). The authors hypothesized that testosterone treatment may directly induce the squamous cell changes and shifts in vaginal flora (20). With under-detection of BV and lack of VMB characterization among transmasculine people, these gaps in knowledge may exacerbate transmasculine health inequities.

Based on the potential effects of exogenous testosterone on VMB, this analysis sought to characterize BV, STI, and HIV testing gaps by comparing testing among the cisgender, transgender, and nonbinary patient populations (all assigned female at birth) in a cohort of primary care patients at an LGBTQ-focused federally qualified health center (FQHC). Based on the very limited knowledge base, we hypothesized that cisgender women patients would have higher BV testing rates than transmasculine patients who take exogenous testosterone.

## 2 Methods

### 2.1 Setting and sample

Howard Brown Health (HBH) is a large FQHC located in Chicago, Illinois, that prioritizes LGBTQ + patient populations. This retrospective cohort was derived from the 2021 electronic health record (EHR) data of Howard Brown Health's primary care patient population (*n* = 26,596) seen at one or more of the eleven HBH clinics located across the city of Chicago. In addition to primary care, HBH offers gynecological care, walk-in STI/HIV testing, behavioral health care, gender-affirming care and surgical navigation, and dental care. While accessible to all sexual orientations, HBH was established in 1974 by a small group of White, cisgender, gay medical students, and it continues to prioritize LGBTQ + populations. From 2011 to 2021, the FQHC's number of unique patients grew fourfold from approximately 6,800–30,000. Of note, the number of transmasculine patients grew eightfold, from approximately 315 to 2,600 over that same period, while the number of cisgender women grew fourfold from 1,450 to 6,230. This analysis relied on 2021 data because in 2022 the FQHC transitioned to a different EHR system, making data extraction for that transitional year challenging. In 2021, characterized as mid- to late-COVID-19 pandemic (21), the FQHC began to offer in-person services again, increasing those visits relative to telehealth, over the course of the year.

The sample was derived by extracting EHR data for all patients with at least one primary care visit in 2021, whether in-person or via telehealth, and who were identifiable in the EHR as assigned female at birth (AFAB) and either transmasculine or a cisgender woman. In this analysis, transmasculine included AFAB patients who identify as men, transgender men, or nonbinary (see Nomenclature section for definitions). Gender identity and sex assigned at birth were patient-reported and collected via intake form, which front desk staff then enter into the EHR. Among all primary care patients, 94% reported a gender (6% either declined or left the field blank), and 86% reported a sex at birth (14% either declined or left the field blank). It is not a standard practice at HBH for providers to update patient demographic data. This analysis excluded patients with a sex at birth of male, intersex, declined, or null, and excluded patients that either declined to report a gender or left gender blank. Gender identity—cisgender women or transmasculine—was the *a priori* primary explanatory variable. Inclusion of transmasculine individuals was restricted to those patients who had a testosterone prescription in their chart in 2021 (*n* = 1,867), 98% of whom had more than one testosterone prescription in 2021, likely reflecting ongoing use.

### 2.2 Data collection

Age in years at first visit was analyzed both continuously (range, mean, standard deviation) and categorically (14–17, 18–24, 25–34, 35–44, 45–54, and 55–64, and 65 and over). Self-reported sexual orientations included gay, bisexual, queer, lesbian, straight, questioning, something else, and declined to answer. Self-reported racial/ethnic categories were Black, White, Latinx/Hispanic, Asian, Native American, Multiracial, Pacific Islander, or unspecified. Insurance was categorized as private, Medicaid, Medicare, sliding scale, or self-pay and other. The final demographic category was geographic region based on patient zip code: Chicago's north side, south side, west side, central Chicago, or outside the city of Chicago.

With respect to gender-affirming care (GAC), specifically hormone therapy (GAHT) that transmasculine patients received, we examined the distribution of testosterone prescriptions by route and dose across demographic characteristics and across BV testing and results. Route was determined using the EHR field for route, prescription description, and instructions, and was categorized into intramuscular, subcutaneous, transdermal (i.e., gel or cream), or other routes (i.e., patch, implant, oral, or unspecified). Dose was determined by the EHR field for dose, prescription description, and instructions. Because GAHT routines vary for patients over time, the first route and dose data in 2021 for each patient were used in analysis. According to established, standardized protocol (22), the route of delivery tends to be one of three for transmasculine patients—intramuscular injection, subcutaneous injection, or transdermal—while the dosing level and frequency often vary depending upon patient transition goals, baseline hormone levels, and comorbidities. Doses were grouped into categories: Initial Low, Initial Typical, and Maximum Typical based on the standardized protocol. Because doses in the EHR covered a wide range, cutoffs for each range were determined as the midpoint between each category listed in the protocol. At a maximum, dosing level and frequency targets stable testosterone and estrogen levels within the standardized range for cisgender men of the same age. Twenty-five percent of transmasculine patients (*n* = 637) had no documentation of a 2021 testosterone prescription in the EHR and were excluded from the final analyses.

Using key words, free text chief complaint data were grouped into five categories: STI/vaginal symptoms, reproductive health, urinary symptoms, gender-affirming care, and other. If a chief complaint contained phrases that qualified for multiple categories, the category was assigned based on the following hierarchy: (1) STI/vaginal symptoms, (2) reproductive health, (3) urinary symptoms, (4) gender-affirming care, and (5) other. Only “other” is mutually exclusive of the other four categories. Data were examined as a patient ever having at least one chief complaint in each category in 2021. Therefore, a single patient can be counted in multiple categories.

Howard Brown Health's clinical protocol for Registered Nurses around BV testing and treatment is as follows: “*Patients presenting with symptoms of abnormal vaginal discharge should be screened utilizing vaginal microscopy for bacterial vaginosis, trichomoniasis, and yeast. A provider should be consulted to review microscopy to confirm diagnosis… Consult with a provider for any presumptive treatment of BV/yeast. If a positive result is confirmed for BV from a BV panel test, the patient should be treated for BV with metronidazole 500* *mg BID × 7 days OR metronidazole gel 0.75% 5* *g/one applicator intravaginally at bedtime for 5 days.”* Aligned with this protocol, we utilized detection of *Gardnerella vaginalis* by real-time PCR, DNA probe (*n* = 1,266) via FDA-approved BD Affirm Bacterial Vaginosis/Vaginitis Panel, as the definition for a BV test.

### 2.3 Statistical analysis

The following data were utilized: demographics, number of primary care visits (categorized as 1 or 2 or more), chief complaint, and testing rates and positive tests for infections related to sexual health: BV, chlamydia, gonorrhea, syphilis, trichomoniasis, and HIV. Chi-square tests were conducted for categorical variables, and Krukshall–Wallace for continuous variables with non-normal distributions (*p* < 0.05). A log binomial regression analysis was conducted to estimate the prevalence risk for receiving a BV test, controlling for demographic characteristics that were unique in bivariate analyses with *p*-value <0.10 (age, sexual orientation, race/ethnicity, insurance type, primary care visits, and geographic region of residence). With cisgender woman as referent, gender was regressed on a binary outcome for having received a test or not in 2021. Data management was conducted in SAS 9.4, and all analyses were conducted in STATA/SE 15. The study protocol was deemed exempt by the FQHC's Institutional Review Board (IRB ID#E-085).

## 3 Results

### 3.1 Sample characteristics of cisgender women compared to transmasculine patients prescribed testosterone in primary care

The demographic characteristics of transmasculine patients using GAHT (*n* = 1,867) and cisgender women patients (*n* = 4,903) who were seen for at least one primary care visit in 2021 are presented in Table 1. On average, cisgender women were older (36.6 vs. 27.6 years) and predominantly identified as straight (62%). Most transmasculine patients identified as transgender men (77.4%) followed by nonbinary (22.6%), while sexual orientation was more varied than the cisgender women patients with 37.2% queer-, 18.8% straight-, and 18.6% bisexual-identified. Cisgender women patients were predominantly Black (40.1%), White (26.9%), or Hispanic/Latina (18.0%), and transmasculine patients were mostly White (58.1%), Hispanic/Latinx (17.2%), or Black (10.2%). More cisgender women were insured by Medicaid (41.7%) than private insurance (30.8%), and more transmasculine patients were privately insured (58.9%) than by Medicaid (22.7%). Finally, cisgender women tended to reside on the North (44.5%) or South (33.7%) Sides of Chicago while most transmasculine patients lived outside of Chicago (49.1%) or on the North Side (35.0%). Supplementary Table S1 compares demographic characteristics of transmasculine patients taking testosterone to those transmasculine patients who were not taking testosterone in 2021. Briefly, the biggest difference was in gender identity, with 71.9% of transmasculine who were not taking testosterone being nonbinary and 28.1% identifying as transgender men, nearly the inverse of transmasculine patients taking testosterone.

**Table 1:** Distribution of characteristics of cisgender women and transmasculine primary care patients seen at least once in 2021 at a Chicago FQHC.

Characteristic	Cisgender women *n* = 4,903 *n* (%)	Transmasculine *n* = 1,867 *n* (%)	*p*-value
Age, categories			*p* < 0.001
≤17	129 (2.6)	38 (2.0)	
18–24	831 (17.0)	776 (41.6)	
25–34	1,879 (38.3)	820 (43.9)	
35–44	848 (17.3)	179 (9.6)	
45–54	522 (10.7)	36 (1.9)	
55–64	436 (8.9)	15 (0.8)	
≥65	258 (5.3)	3 (0.2)	
Sexual orientation			*p* < 0.001
Bisexual	636 (13.0)	347 (18.6)	
Gay	37 (0.8)	145 (7.8)	
Lesbian	355 (7.2)	68 (3.6)	
Queer	332 (6.8)	694 (37.2)	
Questioning	58 (1.2)	41 (2.2)	
Something else	80 (1.6)	127 (6.8)	
Straight	3,041 (62.0)	350 (18.8)	
Declined to answer	364 (7.4)	95 (5.1)	
Race/ethnic identity			*p* < 0.001
Native American	29 (0.6)	9 (0.5)	
Asian American	181 (3.7)	77 (4.1)	
Black	1,966 (40.1)	191 (10.2)	
Hispanic/Latinx	883 (18.0)	321 (17.2)	
Multiracial	141 (2.9)	63 (3.4)	
Pacific islander	20 (0.4)	10 (0.5)	
White	1,319 (26.9)	1,084 (58.1)	
Unspecified	364 (7.4)	112 (6)	
Telehealth visits			*p* < 0.001
Ever	2,025 (41.3)	1,150 (61.6)	
Never	2,878 (58.7)	717 (38.4)	
Insurance type			*p* < 0.001
Medicaid	2,044 (41.7)	423 (22.7)	
Medicare	258 (5.3)	32 (1.7)	
Private	1,508 (30.8)	1,099 (58.9)	
Sliding scale	863 (17.6)	197 (10.6)	
Self-pay	230 (4.7)	116 (6.2)	
Residential region			*p* < 0.001
North side	2,184 (44.5)	653 (35.0)	
South side	1,651 (33.7)	150 (8.0)	
West side	348 (7.1)	121 (6.5)	
Central Chicago	97 (2.0)	27 (1.5)	
Outside Chicago	623 (12.7)	916 (49.1)	

### 3.2 Distribution of transmasculine primary care patients’ testosterone prescriptions by route and dose

**Figure 1:**
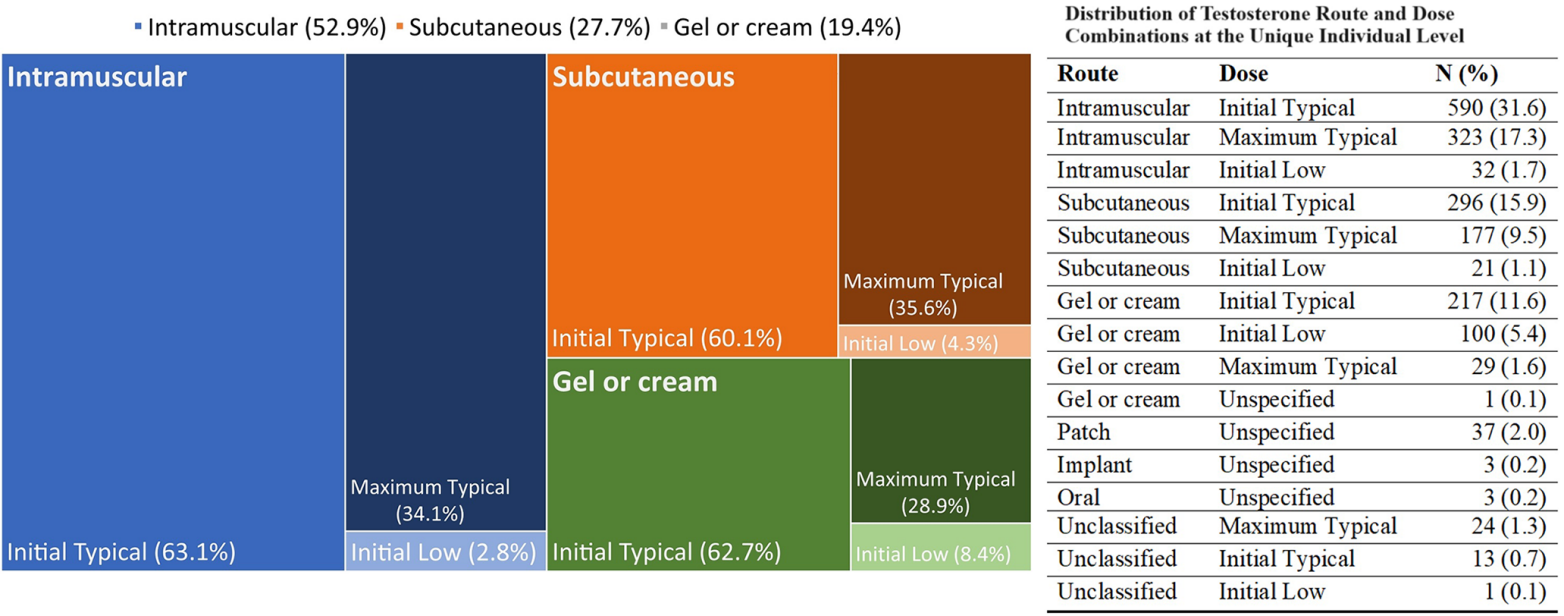
Distribution of testosterone route and dose combinations at the unique individual level. The mosaic plot represents the three main route and dose combinations of testosterone prescription for transmasculine patients at first prescription. The different colors represent the three combinations, with intensity of shading representing the specific regimen, as labelled inside the figure. The numeric frequency distributions, including for uncommon regimens, are shown to the side of the figure.

In terms of GAHT (Figure 1), the majority of transmasculine patients were prescribed testosterone preparations delivered via injections (77.1%)—with more via intramuscular (50.6%) than subcutaneous (26.5%). The remainder were prescribed a cream or gel for transdermal delivery (18.6%), and a minority were prescribed routes via oral, implant, patch, and unclassified (4.3%). The distributions across delivery routes, demographics, and BV testing are shown in Supplementary Table S2, and there were no substantial differences in BV testing or results across routes. Similarly, transmasculine patients showed no substantial differences in BV testing or results by route and dosage (see Supplementary Table S3). Because estrogen is recommended for transmasculine patients experiencing vaginitis (22), we examined BV testing and results in relation to this (Supplementary Table S4). Among 71 (3.8%) of transmasculine patients with an intravaginal estrogen prescription in 2021, 7.0% were tested for BV, as compared to 1.7% of transmasculine patients without an intravaginal estrogen prescription (*p* = 0.010), with no difference in BV positivity by intravaginal estrogen prescription status among those tested.

### 3.3 Chief complaints for cisgender women and transmasculine primary care patient populations

Chief complaint data are summarized in Supplementary Table S5 by gender identity, BV testing, BV results, and sociodemographic characteristics. Cisgender female patients were more likely to have at least one chief complaint for STI/vaginal symptoms than transmasculine patients (25.0% vs. 5.4%), and to have at least one chief complaint for other reproductive health services (19.4% vs. 8.5%). Transmasculine patient visits were predominantly for purposes of gender-affirming care (58.7% vs. 1.8% for cisgender women). Among patients tested for BV (*n* = 884), 70.7% had at least one chief complaint for STI/vaginal symptoms while 29.3% never had a chief complaint for STI/vaginal symptoms. Among those tested but without reported STI/vaginal symptoms, 61.0%, or 158 out of 259 tests, resulted a positive test for BV. Supplementary Table S6 compares BV symptomatic patients to asymptomatic patients stratified by gender identity and summarizes the distribution of BV testing, no testing, positive results, and negative results.

### 3.4 Comparison of BV, STI, and HIV testing rates for cisgender women and transmasculine patient populations

The testing rates and respective case positivity for the two patient groups are summarized in Table 2. In 2021, 17.3% of cisgender women were tested for BV, and 75% of them had positive results. That same year, only 1.9% of transmasculine patients who were using testosterone received a BV test, and among them, 22.2% were positive. When accounting for telehealth visits (see Supplementary Table S7), cisgender women (2.9%) and transmasculine (0.7%) patients who were only seen via telehealth for primary care in 2021 were tested for BV, STIs, and HIV at much lower rates than those patients who had at least one in-person primary care visit. Although BV testing represents the greatest disparity, a far lower proportion of transmasculine patients received tests for STIs and HIV, too. Compared to transmasculine patients using testosterone, cisgender women were also more likely to test positive for chlamydia (10.2% vs. 4.3%), gonorrhea (5.6% vs. 3.4%), trichomoniasis (11.3% vs. 2.3%), syphilis (5.8% vs. 2.0%), and HIV (0.8% vs. 0.2%).

**Table 2:** Distribution of sexual health testing and outcomes of cisgender women and transmasculine primary care patients seen at a Chicago FQHC in 2021.

	Cisgender women *n* = 4,903 *n* (%)	Transmasculine *n* = 1,867 *n* (%)	*p*-value
BV			
Ordered	1,216 (24.8)	75 (4.0)	*p* < 0.001
Tested	977 (19.9)	46 (2.5)	*p* < 0.001
Positive^a^	696 (71.2)	19 (41.3)	*p* < 0.001
Chlamydia			
Tested	2,393 (48.8)	556 (29.8)	*p* < 0.001
Positive^a^	245 (10.2)	24 (4.3)	*p* < 0.001
Gonorrhea			
Tested	2,393 (48.8)	555 (29.7)	*p* < 0.001
Positive^a^	134 (5.6)	19 (3.4)	*p* < 0.04
Trichomoniasis			
Tested	1,520 (31.0)	88 (4.7)	*p* < 0.001
Positive^a^	172 (11.3)	2 (2.3)	*p* < 0.01
Syphilis			
Tested	2,015 (41.1)	595 (31.9)	*p* < 0.001
Positive^a^	116 (5.8)	12 (2.0)	*p* < 0.001
HIV	*n = 4,682*	*n = 1,860*	
Tested	2,008 (42.9)	450 (24.2)	*p* < 0.001
Positive^a^	16 (0.8)	1 (0.2)	*p* < 0.18

^a^ Among those tested and, for HIV, those patients already living with HIV were not counted in the denominator.

### 3.5 Log binomial regression analysis

Results of multivariable log binomial regression analysis adjusted for age, race, insurance status, patient residential area, sexual orientation, and number of primary care visits show that transmasculine patients using testosterone were far less likely to have received a BV test compared to cisgender women [aPR: 0.19 (95% CI: 0.13–0.27), Table 3]. Of note, Black patients and multiracial patients were over two and a half times more likely to have received a BV test compared to White patients (aPR: 2.81 [95% CI: 2.19–3.61], aPR: 2.65 [95% CI: 1.85–3.80], respectively), and Medicaid patients were 1.44 [95% CI: 1.22–1.71] times as likely as privately insured patients. When the telehealth categorical variable was included, the model did not converge (see Supplementary Table S8 for alternative models that included telehealth).

**Table 3:** Crude and multivariable log binomial regression analysis of association between BV testing and gender identity among cisgender women and transmasculine primary care patients in 2021.

Characteristic (reference)	BV tested in 2021	
	Crude PR (95% CI)	aPR (95% CI)
Gender		
Transmasculine vs. cisgender women	0.11 (0.08–0.15)	**0.19** (**0.13–0.27)**
Age (ref: 25–34 years)		
≤17	0.71 (0.45–1.12)	**0.46** (**0.30–0.71)**
18–24	1.02 (0.88–1.19)	1.09 (0.97–1.24)
35–44	0.96 (0.80–1.15)	**0.76** (**0.65–0.89)**
45–54	0.76 (0.59–0.98)	**0.52** (**0.41–0.67)**
55–64	0.54 (0.39–0.75)	**0.35** (**0.26–0.49)**
≥65	0.13 (0.06–0.32)	**0.11** (**0.05–0.27)**
Sexual orientation (ref: Straight)		
Bisexual	0.58 (0.48–0.71)	0.93 (0.78–1.10)
Gay	0.23 (0.12–0.46)	0.87 (0.46–1.66)
Lesbian	0.23 (0.14–0.36)	**0.33** (**0.21–0.52)**
Queer	0.24 (0.18–0.32)	0.75 (0.57–1.00)
Questioning	0.54 (0.30–0.97)	1.03 (0.62–1.68)
Something else	0.23 (0.12–0.44)	0.56 (0.30–1.04)
Declined to answer	0.53 (0.40–0.71)	**0.74** (**0.57–0.97)**
Race/ethnicity (ref: White)		
Native American	0.78 (0.11–5.46)	0.66 (0.10–4.55)
Asian American	1.61 (0.93–2.80)	1.20 (0.70–2.07)
Black or African American	8.22 (6.57–10.30)	**2.81** (**2.19–3.61)**
Hispanic/Latinx	3.13 (2.39–4.10)	**1.89** (**1.45–2.48)**
Multiracial	4.80 (3.29–7.01)	**2.65** (**1.85–3.80)**
Pacific islander	2.97 (0.99–8.86)	2.48 (0.89–6.86)
Unspecified	1.68 (1.10–2.57)	1.15 (0.76–1.76)
Insurance type (ref: Private)		
Medicaid	3.52 (2.95–4.18)	**1.44** (**1.22–1.71)**
Medicare	1.26 (0.81–1.95)	1.07 (0.70–1.62)
Sliding scale	2.92 (2.38–3.58)	**1.64** (**1.35–1.98)**
Self-pay & other	1.81 (1.28–2.56)	1.34 (0.98–1.83)
Primary Care Visits: ≥2 vs. 1	0.83 (0.74–0.94)	1.06 (0.95–1.19)
Residential region (ref: North side)		
South side	5.75 (4.86–6.81)	**2.77** (**2.30–3.34)**
West side	2.71 (2.07–3.54)	**2.07** (**1.60–2.68)**
Central Chicago	1.05 (0.50–2.20)	0.80 (0.39–1.64)
Outside Chicago	1.24 (0.97–1.58)	**1.66** (**1.31–2.11)**

Ref, reference; PR, prevalence ratio; 95% CI, 95% confidence interval.

Bold indicates statistical significance at *p* < 0.05.

## 4 Discussion

Our analysis of 2021 EHR data at a large, LGBTQ-focused FQHC identified marked testing inequities related to the sexual health of transmasculine primary care patients who use testosterone. Controlling for demographic characteristics, transmasculine patients taking testosterone had a nearly 80% lower probability of receiving a BV test compared to cisgender women patients. In keeping with our findings, Pyra and colleagues evaluated HIV and STI testing and diagnosis at HBH across time periods of before, during, and throughout the COVID-19 pandemic (21). They characterized 2021 as a year of transition from mid- to late-stage pandemic, finding that overall testing rates for HBH that year had largely returned to pre-pandemic levels. However, testing and diagnoses among trans and nonbinary populations remained lower than pre-pandemic levels (21). This disparity likely reflects multiple factors and obscures the true positivity rate among transmasculine patients.

Research on transmasculine populations has identified lower PrEP uptake and unique risks for HIV acquisition, but this research is largely in the context of transgender and cisgender men who have sex with men (23, 24). In this context, transmasculine people have indicated that providers are often the critical barrier to sexual healthcare due to an exclusionary focus on cisgender MSM, anti-transgender stigma, a lack of transgender-specific health knowledge, and a limited capacity to meet STI testing needs (10, 25). Our findings suggest additional factors to consider. In the context of the COVID-19 pandemic, access to primary care via telehealth increased, and with more transmasculine patients from outside Chicago seeking gender-affirming care at Howard Brown Health, the frequency of telehealth visits among transmasculine patients increased. However, while our analysis found that BV, STI, and HIV testing rates were greater among cisgender women patients who never had a telehealth visit as compared to those who did, this differential was not observed for transmasculine patients, indicating that barriers other than telehealth format were affecting transmasculine primary care patients. With “opt-out” HIV testing at Howard Brown Health, the disproportionate rate of refusal could not be ascribed to telehealth.

As others have argued, home testing kits are a critical way to mitigate the sexual health disparities experienced by those living in non-urban areas with limited access to care (26). Although Howard Brown Health mailed 14,306 HIV test kits to patients during COVID-19 in 2020, at-home HIV test kits are no longer available. The disproportionate number of transmasculine patients coming from outside Chicago, and correlation of residential region with telehealth visits, indicates a possible lack of access to gender-affirming care in suburban, exurban, and rural areas in Illinois and surrounding states. Novel BV, STI, and HIV testing delivery strategies should be considered and explored with transmasculine patients.

At HBH, all medical providers have completed training to provide gender-affirming care; however, their training and dominant treatment orientations may lend themselves to deprioritize or overlook testing transmasculine patients for a condition like BV—which requires a relatively invasive and potentially gender-disaffirming procedure whereby either the clinician or the patient has to swab the vaginal tract for a specimen. Although care quality for transmasculine patients has advanced primarily in the domain of gender affirmation, especially at HBH, little remains known or understood generally around the microbial impact of testosterone on the VMB. Further, the combination of gender dysphoria around genitalia and the healthcare associated with cisgender women combined with a strong preference for care that affirms their masculinity or nonbinary status, transmasculine patients and their providers alike may grossly ignore or underestimate transmasculine susceptibility to BV or a suboptimal microbial environment in the genital tract. Without understanding the impact of testosterone on transmasculine VMB, providers and patients alike may not have the capacity to recognize the unique symptom presentations of BV or of suboptimal VMB conditions.

Though some studies have highlighted the benefits of administering testosterone replacement therapy via intramuscular injection compared to transdermal or oral routes, they have not included transmasculine individuals (27, 28). Any differential impact that mode of administration has on the composition of the VMB and susceptibility to BV is unknown. In our analysis, approximately 77% of the transmasculine patients with a testosterone prescription were administering it via injection, and 19% were prescribed a topical gel or cream, and with only 1.9% of transmasculine patients receiving a BV test there was no detectable difference in distribution of testing or positivity by route. Future investigation of the impact of testosterone on vaginal symptoms and the VMB will need to be collected prospectively with standardized data capture on route, dose, and frequency of testosterone regimen administration. The findings from prospective, standardized analyses is very relevant for decision-making among transmasculine patients and their providers.

While not perfect vis-à-vis cisgender women, testing and treatment options exist that can reduce BV recurrence and improve VMB composition. For the last 30 years, the “gold standard” BV diagnostic tests have been Amsel's criteria and the Nugent scoring system (29–31). Both these methods have shown low inter-rater reliability, depending upon the clinician or lab technician's skill and experience. A BV diagnosis is additionally challenging vis-à-vis cisgender women due to an etiology that is not well understood; the condition is polymicrobial, lacks a clear definition based on scientific consensus, and is impacted by numerous social, epidemiologic, microbiological, and host factors. In addition, 50%–75% of BV cases may be asymptomatic. New molecular as well as next generation sequencing (NGS) and machine learning approaches to diagnostic tests have been developed and demonstrate higher sensitivity and specificity than Amsel's and Nugent, but have yet to become a new gold standard, and none have been assessed among transmasculine people (7).

Self-sampling for cervical cancer has been demonstrated to be equally effective as provider obtained sampling (32, 33). A self-sampling approach to BV, vaginitis, and STIs may be a critical, patient-centered alternative to current clinician-based testing for transmasculine patients. In cisgender women, a study has shown that self-collected vs. clinician collected samples are comparable for assessing vaginal microbiome composition (34). Studies have established that transmasculine patients prefer self-sampling for HPV vs. clinician-collected Pap smears (35, 36). Self-collected swabs to test for BV in transmasculine patients could improve testing accessibility and acceptability. For all these reasons, studies are needed to establish best practices for community-engaged sexual healthcare for transmasculine people who use testosterone.

Misclassification of transmasculine individuals in the EHR as well as surveillance data is an additional challenge that carries health equity implications (37). If a transmasculine patient's legal sex is male and they indicate male gender rather than “transgender male” in the intake form and do not indicate, or do not have the option to indicate, that the assigned sex at birth was female, that patient may be counted as cisgender male (38) unless a thorough chart review and extensive rule-based algorithm is used to identify transmasculine patients (39). Our analysis relied primarily on discordant gender identity and assigned sex at birth to identify transmasculine patients, and we do not know how many transmasculine patients did not report assigned sex at birth while reporting a cisgender male gender identity. In addition, we excluded 96 charts whereby patients were classified (i.e., likely misclassified) as cisgender women while being prescribed testosterone. We also excluded 108 charts whereby the patients’ sex at birth was female and their gender identity was transgender woman, as these were likely data entry errors in one of the EHR fields. In sum, patients’ varied interpretations of their identities and willingness to disclose them, in addition to the charting variation across providers, may have led to misclassification and exclusion of transmasculine patients from this analysis.

Our analysis has other limitations. The data are not longitudinal and represent a patient cohort from 2021, the mid- to late-COVID pandemic period, where transmasculine testing for HIV and STIs remained lower than pre-pandemic periods. This analysis modeled BV tests with results, but among patients with an order placed for a BV test, transmasculine were more likely to have no corresponding result (52% vs. 30% for cisgender women). This may be due to a number of reasons, one of which could be that laboratories’ computers will not run BV tests for specimens with a legal gender marker of male. We also cannot draw any inferences about the transmasculine BV positivity rate (22.2%) relative to cisgender women's rate (74.5%) since only 1.9% of transmasculine patients were tested compared to 17.3% of cisgender women. The low rate of testing for transmasculine patients may also be due asymptomatic presentation per the BV testing protocol; further, BV molecular diagnostic tests are only recommended for use in symptomatic females (1). Due to variable EHR documentation and lack of full criteria for definitive BV diagnostic approaches, we relied on molecular test orders and results as they were most frequently conducted and consistently reported. Our analysis is derived from EHR data, which are captured for numerous purposes: for billing, patient-centered care, clinical decision-making, and quality improvement and research. EHR fields for capturing prescription route, dose, and frequency medication are unstructured, creating barriers to modeling dosing levels and frequency; structured data elements for GAHT might benefit quality improvement of patient-centered outcomes and health equity research efforts.

## 5 Conclusion

We observed a substantially lower rate of BV, STI, and HIV testing among transmasculine adults as compared to cisgender women in primary care at a large LGBTQ + health center. Rigorous research is needed to address testing inequities for transmasculine people and to understand exogenous testosterone’s impact on vaginal health, including BV and the microbiome. Implementation science frameworks may help advance equitable, patient-centered care and improve health outcomes, including a range of comorbidities not yet well understood in relation to gender-affirming hormonal intervention. Such research should be carried out with engagement of key stakeholders like transmasculine patients, providers, health system leadership, quality improvement staff, and information systems staff to identify determinants of sexual healthcare and testing, and to develop strategies to implement improvements in EHR data capture, clinical procedures, patient processes, and patient-provider communication related to sexual health and gender-affirming care.

## Nomenclature

In this article, the terms cisgender, transgender, transmasculine, and nonbinary are used to differentiate the gender identities of two subpopulations, cisgender women and transmasculine people, derived from a population of patients assigned female at birth (AFAB) and currently eligible for Bacterial vaginosis (BV) testing. Cisgender refers to a gender identity that aligns with the gender associate with one's sex assigned at birth—in this case female. Transmasculine describes both transgender and nonbinary individuals assigned female at birth. Although these concepts of identity and gender are more complex and dynamic than can be captured here, transgender men have a sense of a gendered self that is primarily masculine while nonbinary people identify on a spectrum not uniformly feminine or masculine.

## Data Availability

The datasets presented in this article are not readily available because datasets analyzed for this study are derived from patient records and may only be accessed and analyzed upon request and appropriate data use agreements with corresponding author and Howard Brown Health.
