# Supplementary material for: Bacterial vaginosis testing gaps for transmasculine patients may exacerbate health disparities
Source: Front Reprod Health. 2024 Feb 20;6:1344111. doi: 10.3389/frph.2024.1344111 (PMC10916334; doi:10.3389/frph.2024.1344111)
Supplement: Supplementary file 1 [file Datasheet1.pdf]

Supplementary Material

1 Supplementary Data

Supplementary Material should be uploaded separately on submission. Please include any supplementary data, figures and/or tables.

Supplementary material is not typeset so please ensure that all information is clearly presented, the appropriate caption is included in the file and not in the manuscript, and that the style conforms to the rest of the article.

2 Supplementary Figures and Tables

For more information on Supplementary Material and for details on the different file types accepted, please see here.

Supplementary Table 1. Distribution of characteristics of transmasculine primary care patients seen at least once in 2021 at a Chicago FQHC, comparing those with a testosterone prescription in 2021 to those without one.

Characteristic	Transmasculine (ever T Rx, 2021) n=1,867	Transmasculine (no T Rx, 2021) n=637	p-value
Age			p<0.001
Mean (SD)	27.6 (7.2)	29.8 (8.5)	
Age range	n (%)	n (%)	p<0.001
≤17	38 (2.0)	15 (2.4)	
18-24	776 (41.6)	163 (25.6)	
25-34	820 (43.9)	340 (53.4)	
35-44	179 (9.6)	85 (13.3)	
45-54	36 (1.9)	19 (3.0)	
55-64	15 (0.8)	12 (1.9)	
≥65	3 (0.2)	3 (0.5)	
BV testing			p<0.001
Tested	36 (1.9)	38 (6.0)	
Not tested	1,831 (98.1)	599 (94.0)	
BV results (among tested)			p=0.030
Positive	8 (22.2)	18 (47.4)	
Negative	28 (77.8)	20 (52.6)	
Gender			p<0.001
Transgender males	1,445 (77.4)	179 (28.1)	
Nonbinary	422 (22.6)	458 (71.9)	
Sexual Orientation			p<0.001
Bisexual	347 (18.6)	105 (16.5)	
Gay	145 (7.8)	31 (4.9)	
Lesbian	68 (3.6)	56 (8.8)	
Queer	694 (37.2)	317 (49.8)	
Questioning	41 (2.2)	10 (1.6)	
Something else	127 (6.8)	43 (6.8)	
Straight	350 (18.8)	46 (7.2)	
Declined to answer	95 (5.1)	29 (4.6)	
Race/ethnicity			p<0.001
American Indian	9 (0.5)	2 (0.3)	
Asian	77 (4.1)	17 (2.7)	
Black	191 (10.2)	98 (15.4)	
Hispanic/Latinx	321 (17.2)	98 (15.4)	
Multiracial	63 (3.4)	22 (3.5)	
Pacific Islander	10 (0.5)	2 (0.3)	
White	1,084 (58.1)	329 (51.7)	
Unspecified	112 (6.0)	69 (10.8)	
Insurance Type			p<0.001
Medicaid	423 (22.7)	225 (35.3)	
Medicare	32 (1.7)	10 (1.6)	
Private	1,099 (58.9)	306 (48.0)	
Sliding scale	197 (10.6)	64 (10.1)	
Self pay & other	116 (6.2)	32 (5.0)	
Residential region			p<0.001
North Side	653 (35.0)	346 (54.3)	
South Side	150 (8.0)	79 (12.4)	
West Side	121 (6.5)	65 (10.2)	
Central Chicago	27 (1.5)	11 (1.7)	
Outside Chicago	916 (49.1)	136 (21.4)	

Supplementary Table 2. Distribution of BV testing, positivity, and demographics across testosterone delivery routes for transmasculine primary care patients in 2021.

	Testosterone routes n (%)			
**Characteristic**	Intramuscular	Subcutaneous	Gel or cream	Other/Unclassified
BV testing				
Tested	21 (2.2)	10 (2.0)	4 (1.2)	1 (1.2)
Not tested	924 (97.8)	484 (98.0)	343 (98.9)	80 (98.8)
BV results (among tested)				
Positive	5 (23.8)	2 (20.0)	1 (25.0)	0 (0.0)
Negative	16 (76.2)	8 (80.0)	3 (75.0)	1 (100.0)
Age range				
≤17	15 (1.6)	1 (0.2)	19 (5.5)	3 (3.7)
18-24	390 (41.3)	199 (40.3)	160 (46.1)	27 (33.3)
25-34	418 (44.2)	227 (46.0)	142 (40.9)	33 (40.7)
35-44	102 (10.8)	48 (9.7)	17 (4.9)	12 (14.8)
45-54	14 (1.5)	11 (2.2)	6 (1.7)	5 (6.2)
55-64	6 (0.6)	5 (1.0)	3 (0.9)	1 (1.2)
≥65	0 (0.0)	3 (0.6)	0 (0.0)	0 (0.0)
Sexual orientation				
Bisexual	165 (17.5)	91 (18.4)	77 (22.2)	14 (17.3)
Gay	72 (7.6)	36 (7.3)	25 (7.2)	12 (14.8)
Lesbian	36 (3.8)	14 (2.8)	17 (4.9)	1 (1.2)
Queer	331 (35.0)	190 (38.5)	147 (42.4)	26 (32.1)
Questioning	10 (1.1)	13 (2.6)	14 (4.0)	4 (4.9)
Something else	64 (6.8)	28 (5.7)	27 (7.8)	8 (9.9)
Straight	213 (22.5)	100 (20.2)	23 (6.6)	14 (17.3)
Declined to answer	54 (5.7)	22 (4.5)	17 (4.9)	2 (2.5)
Race/ethnicity				
American Indian	5 (0.5)	3 (0.6)	1 (0.3)	4 (4.9)
Asian	35 (3.7)	21 (4.3)	17 (4.9)	0 (0.0)
Black	111 (11.8)	50 (10.1)	19 (5.5)	11 (13.6)
Hispanic/Latinx	194 (20.5)	68 (13.8)	44 (12.7)	15 (18.5)
Multiracial	30 (3.2)	23 (4.7)	6 (1.7)	4 (4.9)
Pacific Islander	4 (0.4)	3 (0.6)	2 (0.6)	1 (1.2)
White	518 (54.8)	292 (59.1)	232 (66.9)	42 (51.9)
Unspecified	48 (5.1)	34 (6.9)	26 (7.5)	4 (4.9)
Insurance type				
Medicaid	221 (23.4)	115 (23.3)	72 (20.8)	15 (18.5)
Medicare	16 (1.7)	9 (1.8)	5 (1.4)	2 (2.5)
Private	542 (57.4)	281 (56.9)	223 (64.3)	53 (65.4)
Sliding scale	96 (10.2)	59 (11.9)	32 (9.2)	10 (12.4)
Self pay & other	70 (7.4)	30 (6.1)	15 (4.3)	1 (1.2)
Residential region				
North Side	298 (31.5)	185 (37.5)	138 (39.8)	32 (39.5)
South Side	78 (8.3)	37 (7.5)	25 (7.2)	10 (12.4)
West Side	69 (7.3)	27 (5.5)	23 (6.6)	2 (2.5)
Central Chicago	14 (1.5)	5 (1.0)	8 (2.3)	0 (0.0)
Outside Chicago	486 (51.4)	240 (48.6)	153 (44.1)	37 (45.7)

Supplementary Table 3. Distribution of BV testing, positivity, and demographics across top five typical combinations of testosterone delivery route and dosage for transmasculine primary care patients with a testosterone prescription in 2021.

	Top 5 Testosterone Route/Dose Combinations n (%)				
**Characteristic**	IM Initial	IM Maximum	SubQ Initial	Gel/cream Initial	SubQ Maximum
BV testing					
Tested	12 (2.0)	7 (2.2)	7 (2.4)	3 (1.4)	3 (1.7)
Not tested	578 (98.0)	316 (97.8)	289 (97.6)	214 (98.6)	174 (98.3)
BV results (among tested)					
Positive	2 (16.7)	2 (28.6)	1 (14.3)	0 (0.0)	1 (33.3)
Negative	10 (83.3)	5 (71.4)	6 (85.7)	3 (100.0)	2 (66.7)
Age range					
≤17	14 (2.4)	0 (0.0)	1 (0.3)	5 (2.3)	0 (0.0)
18-24	272 (46.1)	105 (32.5)	122 (41.2)	102 (47.0)	67 (37.9)
25-34	242 (41.0)	162 (50.2)	138 (46.6)	92 (42.4)	80 (45.2)
35-44	54 (9.2)	46 (14.2)	22 (7.4)	12 (5.5)	24 (13.6)
45-54	6 (1.0)	6 (1.9)	7 (2.4)	4 (1.8)	4 (2.3)
55-64	2 (0.3)	4 (1.2)	4 (1.4)	2 (0.9)	1 (0.6)
≥65	0 (0.0)	0 (0.0)	2 (0.7)	0 (0.0)	1 (0.6)
Sexual orientation					
Bisexual	97 (16.4)	64 (19.8)	52 (17.6)	44 (20.3)	34 (19.2)
Gay	42 (7.1)	27 (8.4)	24 (8.1)	22 (10.1)	10 (5.7)
Lesbian	28 (4.8)	6 (1.9)	7 (2.4)	8 (3.7)	6 (3.4)
Queer	218 (37.0)	95 (29.4)	116 (39.2)	94 (43.3)	64 (36.2)
Questioning	7 (1.2)	3 (0.9)	9 (3.0)	6 (2.8)	3 (1.7)
Something else	39 (6.6)	24 (7.4)	19 (6.4)	16 (7.4)	9 (5.1)
Straight	119 (20.2)	90 (27.9)	58 (19.6)	19 (8.8)	42 (23.7)
Declined to answer	40 (6.8)	14 (4.3)	11 (3.7)	8 (3.7)	9 (5.1)
Race/ethnicity					
American Indian	3 (0.5)	2 (0.6)	2 (0.7)	0 (0.0)	1 (0.6)
Asian	26 (4.4)	8 (2.5)	16 (5.4)	9 (4.2)	4 (2.3)
Black	63 (10.7)	45 (13.9)	30 (10.1)	13 (6.0)	20 (11.3)
Hispanic/Latinx	114 (19.3)	73 (22.6)	41 (13.9)	28 (12.9)	24 (13.6)
Multiracial	20 (3.4)	7 (2.2)	12 (4.1)	5 (2.3)	10 (5.7)
Pacific Islander	2 (0.3)	2 (0.6)	1 (0.3)	2 (0.9)	2 (1.1)
White	332 (56.3)	175 (54.2)	171 (57.8)	146 (67.3)	108 (61.0)
Unspecified	30 (5.1)	11 (3.4)	23 (7.8)	14 (6.5)	8 (4.5)
Insurance type					
Medicaid	139 (23.6)	75 (23.2)	69 (23.3)	51 (23.5)	43 (24.3)
Medicare	10 (1.7)	5 (1.6)	5 (1.7)	3 (1.4)	4 (2.3)
Private	334 (56.6)	187 (57.9)	173 (58.5)	131 (60.4)	95 (53.7)
Sliding scale	66 (11.2)	27 (8.4)	34 (11.5)	21 (9.7)	21 (11.9)
Self pay & other	41 (7.0)	29 (9.0)	15 (5.1)	11 (5.1)	14 (7.9)
Residential region					
North Side	191 (32.4)	94 (29.1)	110 (37.2)	82 (37.8)	64 (36.2)
South Side	41 (7.0)	29 (9.0)	24 (8.1)	16 (7.4)	12 (6.8)
West Side	48 (8.1)	16 (5.0)	17 (5.7)	10 (4.6)	9 (5.1)
Central Chicago	10 (1.7)	4 (1.2)	2 (0.7)	8 (3.7)	36(1.7)
Outside Chicago	300 (50.9)	180 (55.7)	143 (48.3)	101 (46.5)	89 (50.3)

Supplementary Table 4. Distribution of BV testing and positivity among transmasculine patients prescribed testosterone in 2021, comparing those prescribed intravaginal estrogen in 2021 to those who were not.

**Variable**	**Transmasculine (ever T Rx in 2021)**		
	No intravaginal estrogen Rx, 2021 n=1,796	Intravaginal estrogen Rx, 2021 n=71	p-value
	n (row %)	n (row %)	
BV testing			**0.010**
Tested	31 (86.11)	5 (13.89)	
Not tested	1,765 (96.40)	66 (3.60)	
BV results (among tested)			1.000
Positive	7 (87.50)	1 (12.50)	
Negative	24 (85.71)	4 (14.29)	

Supplementary Table 5. Distribution of BV testing, positivity, and sociodemographics across five categories of chief complaints among cisgender women and transmasculine (with a testosterone

prescription) primary care patients in 2021: STI/vaginal symptoms (Sx), reproductive health, urinary symptoms, gender-affirming care (GAC), and other.

	Chief Complaint Category				
**Characteristic**	**STI/Vaginal Sx n (%)**	**Repo Health n (%)**	**Urinary Sx n (%)**	**HRT/GAC n (%)**	**Other n (%)**
Gender					
Cisgender women	1,226 (25.0)	951 (19.4)	55 (1.1)	90 (1.8)	2,796 (57.0)
Transmasculine	100 (5.4)	158 (8.5)	5 (0.3)	1,095 (58.7)	662 (35.5)
BV					
Tested	625 (70.7)	155 (17.5)	21 (2.4)	22 (2.5)	159 (18.0)
Positive*	482 (75.3)	106 (16.6)	14 (2.2)	7 (1.1)	103 (16.1)
Age range					
≤17	24 (14.4)	8 (4.8)	2 (1.2)	16 (9.6)	119 (71.3)
18-24	377 (23.5)	283 (17.6)	14 (0.9)	543 (33.8)	523 (32.6)
25-34	583 (21.6)	530 (19.6)	21 (0.8)	495 (18.3)	1,236 (45.8)
35-44	190 (18.5)	194 (18.9)	13 (1.3)	105 (10.2)	573 (55.8)
45-54	85 (15.2)	56 (10.0)	3 (0.5)	19 (3.4)	403 (72.2)
55-64	55 (12.2)	35 (7.8)	5 (1.1)	6 (1.3)	361 (80.0)
≥65	12 (4.6)	3 (1.2)	2 (0.8)	1 (0.4)	243 (93.1)
Sexual orientation					
Bisexual	193 (19.6)	196 (19.9)	6 (0.6)	228 (23.2)	431 (43.9)
Gay	14 (7.7)	22 (12.1)	0 (0.0)	92 (50.6)	71 (39.0)
Lesbian	37 (8.8)	65 (15.4)	1 (0.2)	75 (17.7)	260 (61.5)
Queer	94 (9.2)	163 (15.9)	3 (0.3)	398 (38.8)	451 (44.0)
Questioning	19 (19.2)	10 (10.1)	0 (0.0)	25 (25.3)	49 (49.5)
Something else	20 (9.7)	18 (8.7)	1 (0.5)	93 (44.9)	84 (40.6)
Straight	879 (25.9)	566 (16.7)	40 (1.2)	201 (5.9)	1,860 (54.9)
Declined to answer	70 (15.3)	69 (15.0)	9 (2.0)	73 (15.9)	252 (54.9)
Race/ethnicity					
American Indian	5 (13.2)	6 (15.8)	0 (0.0)	9 (23.7)	20 (52.6)
Asian	43 (16.7)	53 (20.5)	2 (0.8)	46 (17.8)	136 (52.7)
Black	722 (33.5)	372 (17.3)	26 (1.2)	139 (6.4)	1,020 (47.3)
Hispanic/Latinx	218 (18.1)	212 (17.6)	6 (0.5)	194 (16.1)	637 (52.9)
Multiracial	41 (20.1)	38 (18.6)	3 (1.5)	39 (19.1)	97 (47.6)
Pacific Islander	6 (20.0)	5 (16.7)	0 (0.0)	5 (16.7)	18 (60.0)
White	222 (9.2)	366 (15.2)	20 (0.8)	666 (27.7)	1,260 (52.4)
Unspecified	69 (14.5)	57 (12.0)	3 (0.6)	87 (18.3)	270 (56.7)
Insurance type					
Medicaid	644 (26.1)	424 (17.2)	30 (1.2)	274 (11.1)	1,241 (50.3)
Medicare	34 (11.7)	22 (7.6)	3 (1.0)	20 (6.9)	221 (76.2)
Private	300 (11.5)	421 (16.2)	7 (0.3)	678 (26.0)	1,344 (51.6)
Sliding scale	288 (27.2)	189 (17.8)	17 (1.6)	146 (13.8)	477 (45.0)
Self pay & other	60 (17.3)	53 (15.3)	3 (0.9)	67 (19.4)	175 (50.6)
Primary care visits					
1	642 (21.3)	228 (7.6)	14 (0.5)	360 (11.9)	1,774 (58.8)
≥2	684 (18.2)	881 (23.5)	46 (1.2)	825 (22.0)	1,684 (44.9)
Residential region					
North Side	404 (14.2)	541 (19.1)	28 (1.0)	377 (13.3)	1,652 (58.2)
South Side	626 (34.8)	298 (16.6)	16 (0.9)	107 (5.9)	849 (47.1)
West Side	106 (22.6)	88 (18.8)	8 (1.7)	91 (19.4)	210 (44.8)
Central Chicago	28 (22.6)	28 (22.6)	1 (0.8)	17 (13.7)	58 (46.8)
Outside Chicago	162 (10.5)	154 (10.0)	7 (0.5)	593 (38.5)	689 (44.8)
*among those tested					

Supplementary Table 6. Distribution of 2021 BV testing and positivity by chief complaint, documented in the EHR, of STI/vaginal symptoms and gender (cisgender women vs. transmasculine (ever T in 2021)).

	**Ever chief complaint of STI/vaginal Sx n (%)**				**Never chief complaint of STI/vaginal Sx n (%)**			
	BV Testing		BV Results		BV Testing		BV Results	
	Tested	Not Tested	Positive	Negative	Tested	Not Tested	Positive	Negative
Gender								
Cisgender women	613 (50.0)	613 (50.0)	478 (78.0)	135 (22.0)	235 (6.4)	3,442 (93.6)	154 (65.5)	81 (34.5)
Transmasculine	12 (12.0)	88 (88.0)	4 (33.3)	8 (66.7)	24 (1.4)	1,743 (98.6)	4 (16.7)	20 (83.3)

Supplementary Table 7. Distribution of sexual health testing and outcomes of cisgender women and transmasculine primary care patients across three categories of primary care visit: never telehealth (tele), ever telehealth, and only telehealth in 2021.

	**Cisgender Women**			**Transmasculine (ever T Rx in 2021)**		
**STI**	**Never Tele**	**Ever Tele**	**Only Tele**	**Never Tele**	**Ever Tele**	**Only Tele**
BV						
Tested	653 (22.7)	195 (9.6)	21 (2.9)	12 (1.7)	24 (2.1)	4 (0.7)
Positive*	508 (77.8)	124 (63.6)	16 (76.2)	3 (25.0)	5 (20.8)	1 (25.0)
Chlamydia						
Tested	1,619 (56.3)	774 (38.2)	148 (20.3)	223 (31.1)	333 (29.0)	84 (15.2)
Positive*	183 (11.3)	62 (8.0)	12 (8.1)	9 (4.0)	15 (4.5)	4 (4.8)
Gonorrhea						
Tested	1,619 (56.3)	774 (38.2)	148 (20.3)	223 (31.1)	332 (28.9)	84 (15.2)
Positive*	105 (6.5)	29 (3.8)	6 (4.1)	4 (1.8)	15 (4.5)	3 (3.6)
Trichomoniasis						
Tested	1,058 (36.8)	462 (22.8)	73 (10.0)	29 (4.0)	59 (5.1)	8 (1.4)
Positive*	127 (12.0)	45 (9.7)	4 (5.5)	0 (0.0)	2 (3.4)	1 (12.5)
Syphilis						
Tested	1,331 (46.3)	684 (33.8)	151 (20.7)	260 (36.3)	335 (29.1)	92 (16.6)
Positive*	87 (6.5)	29 (4.2)	4 (2.7)	6 (2.3)	6 (1.8)	2 (2.2)
HIV	**n=2,770**	**n=1,911**	**n=698**	**n=713**	**n=1,147**	**n=551**
Tested	1,390 (50.2)	618 (32.3)	137 (19.6)	175 (24.5)	275 (24.0)	67 (12.2)
Positive*	12 (0.9)	4 (0.7)	1 (0.7)	1 (0.6)	0 (0.0)	0 (0.0)
*among those tested						

Supplementary Table 8. Alternative multivariable log binomial regression analyses that include telehealth categorical variables, of association between BV testing and gender identity among cisgender women and transmasculine (with a testosterone prescription) primary care patients in 2021.

Characteristic	+Ever telehealth -Region	+Ever telehealth -Primary care visit count	+Only telehealth
	aPR (95% CI)	aPR (95% CI)	aPR (95% CI)
Gender			
Transmasculine vs. cisgender women	**0.22 (0.15-0.31)**	**0.20 (0.14-0.29)**	**0.22 (0.15-0.31)**
Age (ref: 25-34 years)			
≤17	**0.41 (0.26-0.64)**	**0.41 (0.27-0.64)**	**0.43 (0.28-0.67)**
18-24	1.04 (0.92-1.18)	1.04 (0.92-1.18)	1.07 (0.95-1.21)
35-44	**0.77 (0.66-0.90)**	**0.79 (0.67-0.92)**	**0.78 (0.66-0.91)**
45-54	**0.53 (0.42-0.67)**	**0.56 (0.44-0.71)**	**0.54 (0.42-0.69)**
55-64	**0.34 (0.25-0.47)**	**0.37 (0.27-0.52)**	**0.36 (0.26-0.49)**
≥65	**0.10 (0.04-0.24)**	**0.11 (0.05-0.27)**	**0.11 (0.05-0.27)**
Sexual orientation (ref: Straight)			
Bisexual	0.90 (0.76-1.07)	0.93 (0.78-1.09)	0.92 (0.78-1.09)
Gay	0.67 (0.36-1.24)	0.90 (0.48-1.68)	0.90 (0.48-1.68)
Lesbian	**0.34 (0.22-0.53)**	**0.35 (0.23-0.55)**	**0.37 (0.24-0.58)**
Queer	0.77 (0.58-1.03)	0.81 (0.61-1.07)	0.76 (0.58-1.01)
Questioning	0.95 (0.57-1.59)	0.95 (0.59-1.55)	1.02 (0.63-1.65)
Something else	0.56 (0.30-1.03)	0.62 (0.33-1.15)	0.61 (0.33-1.12)
Declined to answer	0.79 (0.60-1.03)	0.78 (0.60-1.02)	0.77 (0.59-1.00)
Race/ethnicity (ref: White)			
American Indian	0.73 (0.11-5.05)	0.66 (0.10-4.53)	0.60 (0.09-4.10)
Asian	1.15 (0.67-1.99)	1.15 (0.67-1.98)	1.16 (0.68-2.00)
Black	**4.25 (3.36-5.38)**	**2.58 (2.01-3.32)**	**2.60 (2.03-3.34)**
Hispanic/Latinx	**2.06 (1.57-2.70)**	**1.84 (1.40-2.41)**	**1.80 (1.38-2.36)**
Multiracial	**3.36 (2.34-4.82)**	**2.65 (1.86-3.78)**	**2.55 (1.79-3.64)**
Pacific Islander	**2.81 (1.01-7.85)**	2.59 (0.96-7.02)	2.19 (0.80-6.03)
Unspecified	1.30 (0.85-1.99)	1.14 (0.75-1.74)	1.18 (0.78-1.79)
Insurance type (ref: Private)			
Medicaid	**1.62 (1.37-1.93)**	**1.44 (1.22-1.71)**	**1.40 (1.18-1.65)**
Medicare	1.24 (0.82-1.87)	1.17 (0.77-1.77)	1.13 (0.75-1.71)
Sliding scale	**1.61 (1.32-1.95)**	**1.54 (1.27-1.86)**	**1.56 (1.29-1.88)**
Self pay & other	**1.47 (1.07-2.02)**	1.36 (1.00-1.86)	1.35 (0.99-1.83)
Primary care visits: ≥2 vs. 1			
≥2	**1.26 (1.12-1.40)**	-	0.99 (0.89-1.11)
Residential region (ref: North Side)			
South Side	-	**2.72 (2.26-3.29)**	**2.72 (2.25-3.28)**
West Side	-	**2.05 (1.59-2.65)**	**2.09 (1.62-2.70)**
Central Chicago	-	0.83 (0.41-1.71)	0.84 (0.41-1.71)
Outside Chicago	-	**1.75 (1.38-2.22)**	**1.80 (1.42-2.27)**
Telehealth visit: ≥1 vs. 0			
≥1 telehealth visit	**0.52 (0.45-0.61)**	**0.59 (0.51-0.68)**	-
In-person visit: 0 vs. ≥1			
0 in-person visits	-	-	**0.21 (0.14-0.31)**
